# Factors Associated with the Recent Increasing Cesarean Delivery Rate at a Japanese Perinatal Center

**DOI:** 10.1155/2013/863282

**Published:** 2013-06-17

**Authors:** Shunji Suzuki, Mariyo Nakata

**Affiliations:** Department of Obstetrics and Gynecology, Japanese Red Cross Katsushika Maternity Hospital, 5-11-12 Tateishi, Katsushika-ku, Tokyo 124-0012, Japan

## Abstract

*Objective*. We examined which specific factors contributed to the increase in Cesarean delivery rate at our hospital over a 10-year period. *Methods*. From January 2002 to December 2012, data on the Japanese singleton deliveries at ≥22-week gestation managed at Japanese Red Cross Katsushika Maternity Hospital were collected. Potential factors associated with the increasing Cesarean delivery rate were selected according to previous studies. In this study, the incidences of intrauterine fetal demise, umbilical artery pH <7.1, and severe perineal laceration were calculated for each year. *Results*. The Cesarean delivery rate at our institution increased significantly during the study period (17.3% in 2002 versus 23.4% in 2012, *P* < 0.01). During the study period, the Cesarean delivery rates in the cases of nulliparity, preterm delivery, low birth weight (<2,500 g), previous Cesarean deliveries and breech presentation were increased significantly. The incidence of intrauterine fetal demise and low umbilical artery pH was significantly decreased, and a negative correlation was found between the Cesarean delivery rate and the incidence of low umbilical artery pH for each year (*r* = −0.92, *P* < 0.01). *Conclusion*. At our institute, the neonatal outcomes seemed to be improved associated with the increased Cesarean delivery rate between 2002 and 2012.

## 1. Introduction

Recently, the Cesarean delivery rate has been reported to be steadily increased in the United States [1–3]. Approximately, one-third of births in the United States are now via Cesarean delivery. The increase has been observed to be among women of all ages and race/ethnicities, in every state, and across all gestational ages. Many theories have been proffered to explain this trend, including a decrease in vaginal births after Cesarean delivery (VBAC), decreased vaginal births of breech presentation, and increased prevalence of high risk pregnancies such as advanced maternal age and some subjective indications during labor such as nonreassuring fetal status and arrest of dilation [1, 2]. Although Cesarean delivery rates that are too low are associated with increased adverse events, Cesarean delivery rates higher than the risk-adjusted expected rate for an institution have not been shown to improve maternal or neonatal outcomes, but they do add cost and unnecessary intervention [4]. Therefore, the examination of Cesarean delivery rate concerning perinatal outcomes is very important for obstetricians.

To date, however, there have not been sufficient observations concerning the Cesarean delivery rate in Japanese populations. In this study, we examined which specific factors contributed to the increase in Cesarean delivery rate at our hospital over a 10-year period.

## 2. Patients and Methods

The protocol for this study was approved by the Ethics Committee of the Japanese Red Cross Katsushika Maternity Hospital. Our hospital is one of the major perinatal centers in Tokyo, Japan (about 1,900–2,000 deliveries per year).

From January 2002 to December 2012, data on the Japanese singleton deliveries at ≥22-week gestation managed at the Japanese Red Cross Katsushika Maternity Hospital were collected. Demographic information and the characteristics of labor were extracted from patient charts to examine the potential factors associated with the increasing Cesarean delivery rate. In this study, the factors were selected according to previous studies [1–3, 5, 6]: nulliparity, advanced maternal age (≥35 years), pregnancy-induced hypertension (PIH), preterm delivery, low birth weight (LBW: neonatal birth weight <2,500 g) and macrosomia (neonatal birth weight ≥4,000 g), history of previous Cesarean deliveries, and breech presentation. PIH was defined as blood pressure ≥140/90 mmHg measured on two or more occasions at least six hours apart with the patient at rest. In our institute, the umbilical cord pH was measured at all deliveries. In addition, we do not perform Cesarean sections for maternal request.

In this study, to examine the effect of the increasing Cesarean delivery rate on the obstetric outcome, the incidences of intrauterine fetal demise (IUFD), umbilical artery pH (UApH) < 7.1, and severe perineal laceration (perineal laceration either third- or fourth-degree laceration) were calculated for each year.

Logistic regression modeling was used to estimate Cesarean delivery rate over time and for each factor. Linear regression was performed to estimate the trend over time for each factor, Cesarean delivery rate for each factor, and obstetric outcomes. Statistical analyses were carried out using the statistical software SAS version 8.02 (SAS Institute, Cary, NC, USA), and differences with *P* < 0.05 were considered significant.

## 3. Results

**Figure 1:**
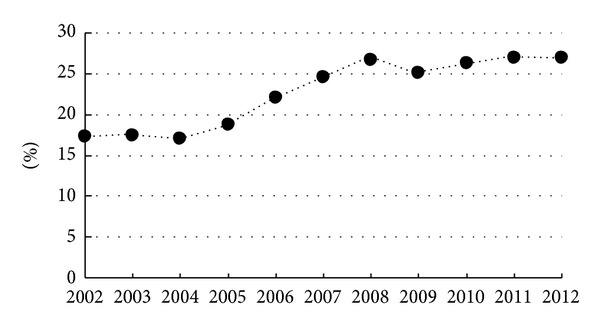
Cesarean delivery rate for each year from 2002 to 2012.

From January 2002 to December 2012, a total of 20,514 Japanese singleton deliveries at ≥22-week gestation were managed at the Japanese Red Cross Katsushika Maternity Red Cross Hospital; 4,086 (19.9%) of those births were delivered by Cesarean section. As shown in Figure 1, the Cesarean delivery rate at our institution increased significantly during the study period (17.3% in 2002 versus 23.4% in 2012, *P* < 0.01). Maternal demographic and obstetric-fetal characteristics among the deliveries, which are potential factors associated with the increasing Cesarean delivery rate, during the study period are presented in Table 1. There was a significant increase in the prevalence of advanced maternal age (≥35 years) during the study period (26.6% in 2002 versus 33.0% in 2012, *P* < 0.01). However, the prevalence of other factors seemed to be stable over time.

**Figure 2:**
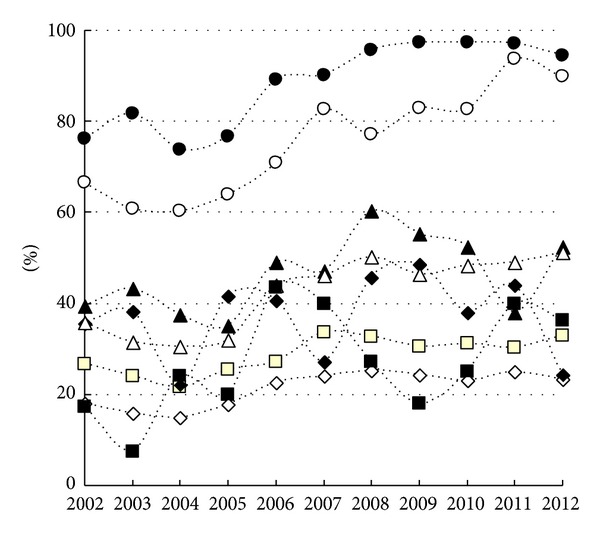
Changes in Cesarean delivery rate by factors (● breech presentation, ○ previous Cesarean deliveries, ▲ preterm delivery, △ neonatal birth weight <2500 g, ■ neonatal birth weight ≥4000 g, □ maternal age ≥35 years, ◆ pregnancy-induced hypertension, and *⋄* nulliparity) from 2002 to 2012.

Figure 2 shows the changes in Cesarean delivery rate for each factor during the study period. As shown in Figure 2, the Cesarean delivery rates with these factors were significantly higher than the average Cesarean delivery rate among all deliveries. During the study period, the Cesarean delivery rates in the cases of nulliparity, preterm delivery, LBW, previous Cesarean deliveries, and breech presentation were increased significantly (nulliparous women: 18.0% in 2002 versus 23.4% in 2012, *P* < 0.01; preterm delivery: 39.5% in 2002 versus 57.5% in 2012, *P* < 0.01; LBW: 35.8% in 2002 versus 57.5% in 2012, *P* < 0.01, previous Cesarean deliveries: 66.7% in 2002 versus 90.0% in 2012, *P* < 0.01; breech presentation: 76.2% in 2002 versus 94.5% in 2012, *P* < 0.01). The Cesarean delivery rates for other factors seemed to be stable over time.

Table 2 shows the changes in the incidence of IUFD, low UApH, and severe perineal laceration during the study period. During the study period, the incidence of IUFD and low UApH were significantly decreased (IUFD: 0.8% in 2002 versus 0.3% in 2012, *P* = 0.03; low UApH: 3.3% in 2002 versus 1.6% in 2012, *P* < 0.01). A negative correlation was found between the Cesarean delivery rate and the incidence of low UApH for each year (*r* = −0.92, *P* < 0.01).

## 4. Discussion

The major findings of the current study are as follows: (1) the Cesarean delivery rate at our institute increased between 2002 and 2012, (2) one of possible reasons for the increasing Cesarean delivery rate is an increase in the prevalence of advanced maternal age (≥35 years), (3) other possible reasons for the increasing Cesarean delivery rate are the increase in Cesarean delivery rates in the cases of nulliparity, preterm delivery, LBW, previous Cesarean deliveries, and breech presentation, and (4) the incidence of low UApH seemed to be improved associated with the increased Cesarean delivery rate. This may be the first report examining the recent changes in Cesarean delivery rate in Japan.

We found the trends in careful correspondences for various high-risk deliveries leading to the increased Cesarean delivery rate at our institute in Japan. These trends seem to be the same as those previously reported in the USA [1–3, 5, 6] and Japan [7–9]. For example, in a recent observation at other perinatal centers in Japan, Cesarean delivery rate was about 50% in primiparous women aged ≥40 years [8].

In our institute, there has been a significant increase in the primary elective Cesarean delivery rate of singleton breech pregnancies due to medical counseling and maternal request between 2000 and 2005 [9]. This phenomenon is associated with the ACOG recommendation in 2001, which recommended Cesarean delivery for the term singleton breech [10]. An increased Cesarean delivery rate in cases with precious Cesarean deliveries seemed to be also an important factor contributing to the current results [1–3, 5]. In our institute, intrapartum asphyxia has been observed to be independently associated with cases of trial of labor after Cesarean delivery (TOLAC) in singleton deliveries beyond 37-week gestation [11]. The asphyxia seemed to be associated with failed vacuum-extraction and/or forceps delivery during TOLAC. Therefore, our previous findings may be encouraging for the counseling of patients regarding a possible attempt at TOLAC [11]. From our impressions, these trends in delivery modes of cases with previous Cesarean deliveries and those with breech presentation have been expanded to all areas of Japan.

Unfortunately, there are some limitations in this study with the lack of taking into consideration confounders such as education/social economic status/immigrant status/antenatal cares access, all variables known to affect mode of delivery [1–3, 5, 6]. In addition, we believe that “defensive medicine” and litigation as an explicit incentive for performing Cesarean section are unlikely to play a large role. In this study, the increased Cesarean delivery rate seemed to contribute to the improvement of neonatal outcomes such as the decreased low UApH. Therefore, it appears that on a global level, the birth process is becoming more medicalised and it may need the increasing in Cesarean delivery rate in Japan. On the other hand, the increasing in Cesarean delivery rate in the past has contributed to the reduction in adverse events; however, the impact of the increasing in Cesarean delivery rate on the maternal and/or neonatal outcomes further from now cannot be predicted. Therefore, in the future, we have to pay attention be the changes in Cesarean delivery rate and perinatal outcomes at our institute in Japan.

In conclusion, the Cesarean delivery rate at our institute increased between 2002 and 2012. The incident of low UApH seemed to be improved associated with the increased Cesarean delivery rate.

## Figures and Tables

**Table 1:** Demographic and obstetric characteristics in singleton pregnancies delivered at ≥22-week gestation.

Year	2002	2003	2004	2005	2006	2007	2008	2009	2010	2011	2012	Total
*N *	1733	1747	1912	1873	1873	1903	1960	1957	1987	1817	1752	20514
Nulliparity	929 (53.6)	953 (54.6)	1002 (52.4)	934 (47.9)	933 (49.8)	960 (50.4)	959 (48.9)	973 (49.7)	990 (49.8)	877 (48.3)	893 (51.0)	10403 (50.7)
Maternal age ≥ 35 years	349 (20.1)	353 (20.2)	492 (25.7)	501 (26.7)	562 (30.0)*	553 (29.1)*	682 (34.8)*	686 (35.1)*	731 (36.8)*	676 (37.2)*	624 (35.0)*	6209 (30.3)
Pregnancy-induced hypertension	90 (5.2)	94 (5.4)	99 (5.2)	106 (5.7)	106 (5.7)	92 (4.8)	91 (4.6)	105 (5.4)	95 (4.8)	91 (5.0)	115 (6.6)	1084 (5.3)
Previous Cesarean deliveries	147 (8.5)	151 (8.6)	154 (8.1)	195 (10.4)	152 (8.1)	178 (9.4)	197 (10.1)	206 (10.5)	224 (11.3)	210 (11.6)	190 (10.8)	2213 (0.9)
Breech presentation	42 (2.4)	44 (2.5)	46 (2.4)	47 (2.5)	46 (2.5)	44 (2.3)	45 (2.3)	38 (1.9)	38 (1.9)	36 (2.0)	37 (2.1)	463 (2.3)
Preterm delivery	162 (93)	141 (8.1)	181 (9.5)	199 (10.6)	165 (8.8)	164 (8.6)	172 (8.8)	170 (8.7)	193 (9.7)	177 (9.7)	181 (10.3)	1905 (9.3)
Neonatal birth weight < 2500 g	226 (13.0)	239 (13.7)	285 (14.9)	288 (15.4)	271 (14.5)	236 (12.4)	280 (14.3)	256 (13.1)	290 (14.6)	271 (14.9)	270 (15.4)	2912 (14.2)
Neonatal birth weight ≥ 4000 g	23 (1.3)	27 (1.5)	25 (1.3)	20 (1.1)	16 (0.9)	20 (1.1)	11 (0.6)	11 (0.6)	20 (1.0)	15 (0.8)	11 (0.6)	199 (1.0)

Data are presented as numbers (percentage).

*P < 0.05 versus 2002.

**Table 2:** Changes in perinatal outcomes for each year from 2002 to 2012.

Year	2002	2003	2004	2005	2006	2007	2008	2009	2010	2011	2012	Total
*N *	1733	1747	1912	1873	1873	1903	1960	1957	1987	1817	1752	20514
Cesarean deliveries	300 (17.3)	304 (17.4)	327 (17.1)	352 (18.8)	413 (22.1)	469 (24.6)	523 (25.1)	513 (25.1)	522 (26.3)	491 (27.0)	472 (26.9)	4686 (22.8)
Intrauterine fetal demise	14 (0.8)	13 (0.7)	14 (0.7)	11 (0.6)	11 (0.6)	5 (0.2)*	4 (0.2)*	5 (0.3)*	5 (0.3)*	3 (0.2)*	5 (0.3)*	90 (0.4)
Umbilical artery pH < 7.1	58 (3.3)	53 (3.0)	67 (3.5)	55 (2.9)	59 (3.2)	40 (2.1)	37 (1.9)*	29 (1.5)*	27 (1.4)*	30 (1.7)*	28 (1.6)*	468 (2.3)
Severe perineal laceration	15 (0.9)	14 (0.8)	15 (0.8)	17 (0.9)	15 (0.8)	14 (0.7)	16 (0.8)	18 (0.9)	16 (0.8)	15 (0.8)	15 (0.9)	170 (0.8)

Data are presented as numbers (percentage).

*P < 0.05 versus 2002.
